# Efficacy of nonselective optogenetic control of the medial septum over hippocampal oscillations: the influence of speed and implications for cognitive enhancement

**DOI:** 10.14814/phy2.13048

**Published:** 2016-12-06

**Authors:** Benjamin J. Blumberg, Sean P. Flynn, Sylvain J. Barriere, Philippe R. Mouchati, Rod C. Scott, Gregory L. Holmes, Jeremy M. Barry

**Affiliations:** ^1^ Department of Neurological SciencesUniversity of Vermont College of MedicineBurlingtonVermont; ^2^ Department of Neurology, Institute of Child HealthUniversity College LondonLondonUnited Kingdom

**Keywords:** Cognition, gamma, optogenetics, oscillations, theta

## Abstract

Optogenetics holds great promise for both the dissection of neural circuits and the evaluation of theories centered on the temporal organizing properties of oscillations that underpin cognition. To date, no studies have examined the efficacy of optogenetic stimulation for altering hippocampal oscillations in freely moving wild‐type rats, or how these alterations would affect performance on behavioral tasks. Here, we used an AAV virus to express ChR2 in the medial septum (MS) of wild‐type rats, and optically stimulated septal neurons at 6 Hz and 30 Hz. We measured the corresponding effects of these stimulations on the oscillations of the MS and hippocampal subfields CA1 and CA3 in three different contexts: (1) With minimal movement while the rats sat in a confined chamber; (2) Explored a novel open field; and (3) Learned and performed a T‐maze behavioral task. While control yellow light stimulation did not affect oscillations, 6‐Hz blue light septal stimulations altered hippocampal theta oscillations in a manner that depended on the animal's mobility and speed. While the 30 Hz blue light septal stimulations only altered theta frequency in CA1 while the rat had limited mobility, it robustly increased the amplitude of hippocampal signals at 30 Hz in both regions in all three recording contexts. We found that animals were more likely to make a correct choice during Day 1 of T‐maze training during both MS stimulation protocols than during control stimulation, and that improved performance was independent of theta frequency alterations.

## Introduction

The physiological capacity to temporally coordinate dynamic neural activity within and between neural networks is believed to underlie normal cognitive processes (Fenton 2015; Barry et al. 2016). Data supporting this theory largely stem from the demonstration that the temporal coordination of neuronal firing, with respect to theta oscillations within the hippocampal circuit (Mizuseki et al. 2009), is correlated with learning and memory (Robbe and Buzsaki 2009; Douchamps et al. 2013; Siegle and Wilson 2014; Barry et al. 2016). Specifically, both modeling and experimental work suggest that the dynamic phase relationships of synaptic current as well as the timing of action potentials during theta rhythm are critical in both encoding and retrieval by organizing the transfer of neural information between the hippocampus and neocortex and within the hippocampal circuit (Hasselmo 2005; Siegle and Wilson 2014). The temporal discoordination of CA1 place cell action potentials with respect to local theta oscillations was also found to correlate with learning deficits on a complex spatial task (Barry et al. 2016).

Decades of research has begun to unravel the complex physiological mechanisms that underpin the generation of the theta rhythm along the septo‐hippocampal axis (Green and Arduini 1954; Petsche et al. 1962; Freund and Antal 1988; Stewart and Fox 1989; Stewart and Fox 1990; Dragoi et al. 1999; Buzsaki 2002; Wang 2002; McNaughton et al. 2006aa; Vandecasteele et al. 2014; Bender et al. 2015, 2016; Mamad et al. 2015; Tsanov 2015; Gangadharan et al. 2016) that ultimately provides the temporal framework for the coordinated suppression or facilitation of synaptic inputs (Csicsvari et al. 1999) across the anatomical expanse of the hippocampal formation (Lubenov and Siapas 2009). Recent developments in the capacity to manipulate hippocampal oscillatory activity through optogenetic manipulation of the septal pacemaker (Laxpati et al. 2014; Vandecasteele et al. 2014; Bender et al. 2015; Mamad et al. 2015) demonstrate the potential of this approach to serve as both a powerful tool for addressing fundamental basic science questions regarding the role of theta in learning and memory (Buzsaki 2002; Hasselmo 2005) and function as a neuroprosthetic that generates artificial oscillations in those with learning and memory difficulties. However, before researchers can address these possible uses of novel technology, there are two critical obstacles that must be addressed. The first is that attempts to artificially generate oscillations have met with mixed results, depending largely on learning and memory demands (Turnbull et al. 1994; McNaughton et al. 2006aa; Shirvalkar et al. 2010; Lipponen et al. 2012) and few studies have examined how intrinsic oscillations might integrate with artificial oscillations in relation to current behavior (Bender et al. 2015). The second obstacle is that although cell‐type selectivity in optogenetics has done much for the dissection of the septo‐hippocampal circuit, this largely necessitates the use of transgenic mouse lines. As transgenic rat lines are still rare (Mamad et al. 2015), the method is largely unavailable to those that prefer rats as a model organism in cognitive research or for use in paradigms that require rat models (Iannaccone and Jacob 2009). Previous studies have shown that it is possible to entrain hippocampal oscillations through optogenetic control of the medial septum (MS) in wild‐type rats (Laxpati et al. 2014), although the efficacy of this entrainment in relation to movement, which has been shown to be an important variable in cell‐selective optogenetics (Vandecasteele et al. 2014; Bender et al. 2015), has not been shown. We therefore carried out several experiments to test the efficacy of nonselective optogenetic control of the medial septum in the entrainment of oscillations in both CA1 and CA3 subfields of the dorsal hippocampus while rats were in three different behavioral contexts: (1) Limited motor activity in a narrow ceramic chamber; (2) Freely exploring a novel open field; and (3) Learning a hippocampal‐dependent memory task.

In agreement with previous work, we show that nonselective optogenetic theta stimulation of the medial septum can clearly alter theta oscillations in the hippocampus. While the 1:1 entrainment of theta oscillations is possible during 6 Hz stimulation, at least in CA3, the stimulation interacts with ongoing intrinsic oscillations in a complex manner that appears to depend largely on the animal's mobility and speed. In contrast, gamma range optogenetic septal stimulation at 30 Hz was found to robustly increase the amplitude of corresponding frequency ranges in both hippocampal regions in all three recording contexts. Finally, we demonstrate for the first time that rather than interfering with memory processes, both stimulation protocols improve performance during training on a T‐maze task independently of theta frequency alterations.

## Methods

### Overview

**Figure 1:**
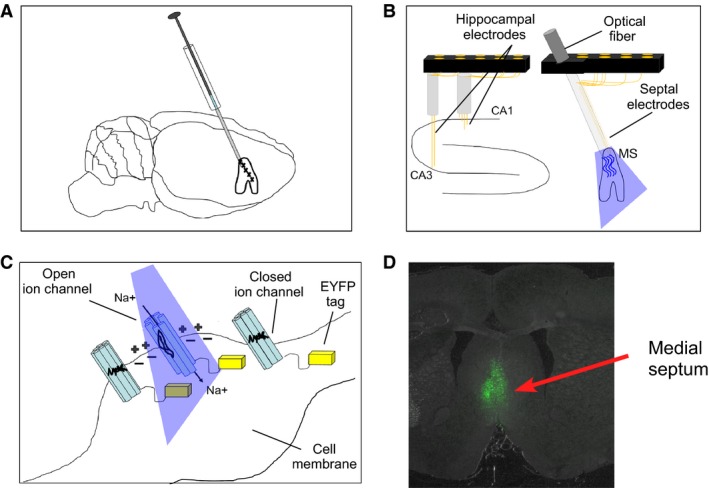
Methodology of AAV injection containing channelrhodopsin (ChR2) into the medial septum, optical stimulation, hippocampal, and medial septum electrophysiology: (A) ChR2 was delivered by AAV injection into the medial septum of adult rats. Expression of ChR2 in the pace‐making cells of medial septum allows for the optical control of septal oscillations; (B) Arrangement of optical probe and EEG recording wires in the medial septum as well as EEG recording wires in both CA1 and CA3 fields of the dorsal hippocampus; (C) In response to blue light, ChR2‐expressing neurons undergo a conformational change leading to the opening of cation channel pores and the conductance of positively charged ions such as Na^+^. The C‐terminal end of ChR2 extends into the intracellular space and is replaced by yellow light‐sensitive proteins (EYFP indicated by yellow blocks) that were used for visualizing the morphology of ChR2‐expressing cells shown in D; (D) Histological example of forebrain tissue section under yellow light. Green fluorescence indicates cells in the medial septum expressing ChR2.

Eight adult male wild‐type Sprague–Dawley rats received medial septum injections of AAV (adeno‐associated virus) that expressed channelrhodopsin. Two weeks post injection, rats were chronically implanted with an optic fiber with an attached recording electrode in the medial septum. Recording electrodes were implanted bilaterally in each hippocampus in both regions CA1 and CA3 (See Fig. 1). Following recovery, rats were stimulated with yellow light control and blue light frequencies at 6 Hz and 30 Hz while the animals either had limited mobility in a narrow ceramic chamber, explored a novel environment, or learned a T‐maze behavioral task.

All procedures were approved by the University of Vermont animal care and use committee and conducted in accordance with guidelines from the National Institutes of Health.

### Optical fiber preparation

We used a 200‐*μ*m multimode optic fiber (Thorlabs, CFLC230‐10; Montreal, Canada) as part of our chronic implant in order to allow for light stimulation of the medial septum. We stripped 25–35 mm of optic fiber using a microstripper. Using a razor blade, we stripped fiber from the main spool, leaving ~10 mm of unstripped fiber. We scored the end of the stripped fiber with a diamond knife on all sides. Hemostats were attached to opposite ends of the optic fiber and pulled apart. The piece of scored and stripped optic fiber then broke off cleanly. The optic fiber was then glued to a 230‐*μ*m ferrule (Thorlabs, CFLC230‐10; TP01235931). First, the ferrule was placed in a vice with the convex side facing down. A droplet of dark epoxy (Precision Fiber Products, Epoxy 353ND/8 oz kit; Part A – PB117651), Part B PB117685) was then added to the larger (upward facing) end of the ferrule, so that there was an outward bubble. The remaining fiber covering was removed and the previously unstripped end was then placed through the epoxy, leaving 15 mm of fiber from the larger end of the ferrule.

Using a heat gun, the dark epoxy was heated until cured (30 secs–1 min). The fiber was removed from the vice and the ferrule was held with a hemostat. All four sides of the fiber were then scored at the convex end of the ferrule using a diamond knife. To increase light transmittance, this cut end was polished using a fiber polishing kit (Thorlabs, Fiber polishing/lapping film for use with ceramic ferrules). Five different grades of polishing paper were used (30 *μ*m, 6 *μ*m, 3 *μ*m, 1 *μ*m, 0.02 *μ*m grit) on a polishing glass plate/silicone pad (THORLABS, CTG913/NRS913A). While the ferrules were held with hemostats, the cut end of the fiber was polished at each grade of polishing paper. Finally, the percent transmittance of light through the fiber was tested using 100% blue light transmittance generated from Spectralynx light‐emitting diode (LED) source and measured by a light meter with a photodiode sensor (Thorlabs; Model PM100D). Using a 50‐*μ*m patch cable for testing, only fibers that allowed for at least 70% light transmittance at approximately 0.5 mm from the tip of the optical fiber were used in our implants.

### Viral injection and chronic implantation surgeries

Two separate surgeries were carried out on adult male Sprague–Dawley rats (*n* = 8) that were separated by 2 weeks. Rats were anesthetized with inhaled isoflurane and placed in a stereotaxic frame where all stereotaxic coordinates were relevant to Bregma (Paxinos and Watson 1998).

Viral injection: The skull was exposed and a burr hole was placed in the skull (AP = 0.7 mm; ML = −1.4 mm) that allowed for targeting access of the vertical limb of the diagonal band of Broca of the medial septum with a Hamilton injection syringe (Fig. 1A). The syringe was inserted into the brain at 12° and placed at a final depth of 7.1 mm from brain surface. A total volume of 0.95 *μ*L of adeno‐associated virus expression of humanized ChR2 with H134R mutation fused to EYFP driven by human synapsin I promoter (AAV2‐hSyn‐hChR2(H134R)‐EYFP; 5.7 × 10^12^ virus molecules/mL; UNC Vector Core, Chapel Hill, NC) was injected into the medial septum at a rate of 0.1 *μ*L/min. The first injection of 0.15 *μ*L was made at 7.1 mm. The needle was then retracted three times at 0.3 mm steps with injections of 0.2 *μ*L, 0.25 *μ*L, and 0.2 *μ*L at each consecutive step. In the final step, the needle was raised 0.2 mm to a final depth of 6.0 mm and an ultimate injection of 0.15 *μ*L was made. The wound was sutured and the rats were returned to their home cages to recover.

Prior work has shown that AAV virus with the hSYN promoter nonselectively transfects different cell types in the medial septum (Chiruvella 2015). While a majority of the transfected cells were found to be glutamatergic or GABAergic (approximately, 70% of each cell type), a minority of cholinergic cells were also transfected (6%).

Hippocampal and septal implants: Two weeks post injection, two custom electrode arrays (Grasshopper Machine Works, New Hampshire, USA) were implanted in both the medial septum and the dorsal hippocampus (Fig. 1B). The medial septum implant included an optic fiber with an array of eight recording electrodes glued to the surface that extended 0.25–0.5 mm from the end of the optic fiber. The optical/recording ensemble was lowered into the medial septum along the same path previously taken by the Hamilton injection syringe, with the end of the optical fiber lowered to a final depth of ~6.2 mm below brain surface. The hippocampal implant consisted of eight 50‐*μ*m diameter stainless steel EEG electrodes (California Fine Wire, CA, USA) that were placed in both the left and right hippocampus and were arranged so that medial electrodes targeted CA1 (AP = −3.7 mm; ML = ±2.5 mm; DV = 2.5–2.8 mm) and lateral electrodes targeted CA3 (AP = −3.7 mm; ML = ±3.8 mm; DV = 3.7–4.0 mm). Four skull screws (FHC Inc.) were inserted, two were anterior to bregma, while the two remaining screws were placed over the left and right of the cerebellum. Grounding was achieved via connection to the right cerebellar screw while a reference wire was placed through a small burr hole at brain surface over the cerebellum. Both implants were fixed to the skull via the skull screws and Grip Cement (Dentsply Inc.). The wound was sutured and topical antibiotic was applied. The interval between surgery and the beginning of electrophysiological recording was 1 week.

### Stimulation and recording protocols

A 200‐*μ*m multimode optic fiber (Thor Laboratories, Budapest, Hungary) was used to connect the implanted optic fiber's 1.25 ceramic ferrule to Spectralynx, a computer‐controlled optical LED system (Neuralynx, Montana, USA). Light intensity was set to 100% and transmitted intensity into the medial septum ranged from 1.4 to 1.8 mW. The pulse program (Neuralynx, Montana) was used to create protocols that generated square wave pulses at two stimulation frequencies of 6 Hz (on/off at 83.3 msec pulses) and 30 Hz (on/off at 16.6 msec pulses). We settled on 6 Hz and 30 Hz stimulation frequencies as pilot studies revealed that resultant matching hippocampal oscillations were visibly evident in the raw EEG. In addition, we chose these optical stimulation frequencies as they fall into functionally relevant hippocampal theta and gamma bandwidths. The theta frequency is the largest amplitude oscillation in hippocampus, associated with exploratory behavior and is principally generated by entorhinal cortical inputs into the distal apical dendrites of pyramidal cells (Buzsaki 2002). The waxing and waning of rhythmic inhibition corresponds to membrane potential fluctuations resulting in the temporal synchronization of neural activity on a ~140 msec timescale. The sequence of theta phase is therefore associated with the alternation of increased and decreased discharge probability of hippocampal neurons across the septotemporal axis (Fox 1989; Ylinen et al. 1995). In contrast, gamma oscillations are locally generated by the ongoing interaction of pyramidal cells and fast spiking interneurons in which pyramidal cells excite basket cells which then in turn inhibit pyramidal cells (Buzsaki and Wang 2012). Theta has been found to be necessary for spatial memory performance (Winson 1978) and is associated with the segmenting of spatial experience and modulated by behavior and cognitive demand (Gupta et al. 2012) as well as the formation and segregation of neuronal assemblies (Buzsaki 2002). Moreover, gamma oscillations have been hypothesized to “route” information flow in CA1 (Colgin et al. 2009) where slow gamma is more strongly coupled to CA3 and believed to be essential for memory storage (Steffenach et al. 2002).

The rat's location in the arena was sampled using a digital camera that detected a LED placed near the animal's head. This tracking information was filtered and recorded utilizing custom software (Neuralynx, Montana, USA) that allowed for the synchronization of the rat's position and speed with properties of the recorded EEG signals.

Rats were tethered to a recording cable during recording sessions in all contexts. LFP signals were preamplified ×1 at the headstage and channeled through the tether cable to the signal amplifiers and computer interface. LFPs (Local Field Potentials) were filtered at 1–9000 Hz (Neuralynx, Montana). All signals were referenced against a 50‐*μ*m diameter stainless steel wire (California Fine Wire, CA, USA) placed at brain surface over the cerebellum.

#### Narrow ceramic chamber

The purpose of the narrow chamber experiment was to test the efficacy of nonselective optogenetic septal control over hippocampal oscillations in the absence of exploratory movement or cognitive demand. Recording sessions were made while rats rested in a 40 cm high ceramic flower pot that was 27 cm wide at the base and lined with home cage bedding. The size of the ceramic pot limited the rat's movements to rearing and minor head movements and therefore limited the amount of theta oscillations associated with active exploration. Blue light stimulation protocols at 6 and 30 Hz consisted of five rounds of stimulations each. Each stimulation lasted 10 sec, with an interstimulus interval of 20 sec.

#### Novel environment

The purpose of the novel environment experiment was to test the efficacy of septal control over hippocampal oscillations during active exploration and examine the interaction of artificially generated oscillations with intrinsic oscillations. Animals were recorded for 20‐min sessions during exploration of three consecutive novel environments. Stimulation protocols in the first environment consisted of yellow light control, while stimulation in the second and third novel environments consisted of blue light stimulation at 6 Hz and 30 Hz consecutively. Stimulations began as soon as the animals were introduced into the arena and continued until the end of the 20‐min session. The same 76 cm diameter gray cylinder was used in three different rooms. A different cue card covering 60° of arc was used in each environment that consisted of a white card with black stripes in vertical, horizontal, or diagonal orientations. Similar protocols have been demonstrated to initiate complete remapping of hippocampal place cells, and by inference, considered by the rats to be novel environments (Leutgeb et al. 2005).

#### T‐ maze

The purpose of the T‐maze alternation experiment was to test for the effects of MS optical stimulation on the acquisition and performance of a cognitive task. Following 1 week of food deprivation, the animals were trained on the T‐maze alternation task (Deacon and Rawlins 2006). The T‐maze consisted of a start box (20 cm × 15 cm), running lane (118 cm × 15 cm), and goal arms (137 cm × 15 cm). The animals were initially habituated in the T‐maze without food reward and then placed in the maze with 20 mg food pellets (BioServ; Flemington, NJ) scattered throughout the maze. Food pellets were gradually moved to only the goal arms. Once the rats readily ran from the start box to the goal arms, three food pellets were placed in metallic food cups that were 3 cm in diameter and approximately 5 cm from the end of each goal arm. The animals were trained for 5–10 trials in order to be certain that they visited both arms. One goal arm was then randomly baited while access to the opposite arm was blocked. Once animals learned to go to both arms, they were considered ready for testing.

During the sampling trial, the rat was first placed in the start box. The gate was opened and the rat ran to the end of the runway. For the first run, the rat was forced to go into the open arm with the food award. On the choice trial, both arms were open and the rat was rewarded with food if it went into the previously unrewarded arm. After reward, the animal was immediately returned to the start box. This sequence (sample and choice) was repeated for 30 test trials. Stimulations with 6 Hz yellow light and 6 Hz and 30 Hz blue were continuously applied from the start box to the completion of the task. The order of stimulations was randomized and consisted of 10 control, 10 6 Hz, and 10 30 Hz trials, so as no three stimulations of the same type were repeated in a row. The order of left and right choice directions was also randomly determined. The stimulation began before the start of the sample run and was not turned off until after the animal reached the end of a goal arm during the choice run. The animals were then retested in the T‐maze. The first 30 trials on Day 1 of training were then compared to the 30 trials on Day 2 in order to test for the effects of stimulation on learning and memory.

### Signal processing

Spectrograms were calculated with Matlab Spectrogram (window = SF/2, overlap = SF/2.4: SF = sampling frequency) and were used to calculate measures of signal amplitude and frequency at 5–12 Hz and 28–32 Hz bandwidths. In order to take into account the possibility of 6 Hz stimulation effects in a smaller theta bandwidth, we also analyzed “theta‐lo” signal properties in the 5–7 Hz range.

#### Mean spectrums

For narrow ceramic chamber recordings, individual mean spectrums consisted of the mean of all spectrums for 10‐sec epochs before stimulation and 10‐sec epochs during stimulation. For novel environment recordings, data from the mean spectrum were calculated for the entire 10‐min recording session during control yellow light and blue light stimulations at both 6 Hz and 30 Hz. Mean spectrums in blue light and yellow light control sessions were then compared using paired t‐tests. With regard to recording sessions in the T‐maze, mean spectral data were calculated for each pass the animal made through the center arm of the T‐maze on both training days. As described below in the statistical methods, the spectral data for multiple runs were then clustered by individual rat.

#### Peak‐theta frequency

Using a 1‐Hz wide moving window (moving at 0.1 Hz), the average amplitude of theta in the 1‐Hz window was calculated across the theta range (5–12 Hz) for each stimulation period. The midpoint of a 1‐Hz moving window with the greatest average amplitude was designated as the peak‐theta frequency and the average amplitude for that window was designated peak theta.

#### Speed/theta

We analyzed the linear relationship between animal speed in relation to theta frequency and amplitude in a similar manner to previous work (Richard et al. 2013) for data collected in the novel environment context.

### Statistical analyses

As the behavioral and EEG dataset from narrow ceramic chamber and during the T‐maze contain data from multiple behavioral choices or stimulations in single animals, the assumptions of independence of observations are invalid. The observations for each of these measures within single animals are likely to be correlated and these data can be represented as a cluster. In this case, the existence of a relationship between each measure of interest within an individual animal may then be assumed (Ziegler et al. 1998). In this study, we used GEE (SPSS; Armonk, NY), a class of regression marginal model, for exploring multivariable relationships between clustered signal property data for individual animals sorted by stimulation type in each recording context. At the behavioral level, we tested for differences in correct choices on both Day 1 and Day 2 during optical stimulation protocols of 6 Hz and 30 Hz. With regard to EEG, we tested the average response to the frequency and amplitude of oscillations in the theta range (5–12 Hz) as well as the amplitude of oscillations between 28 and 32 Hz during both 6‐Hz and 30‐Hz blue light optical stimulation protocols compared with yellow light controls or prestimulation epochs.

## Results

### Narrow ceramic chamber

Seven rats were recorded in the narrow ceramic chamber during 6 Hz and 30 Hz blue light stimulation protocols of the medial septum. Responses in the medial septum and corresponding changes in the hippocampus were not found with two of these animals at either frequency. Histology showed that the optical fiber for one of these animals was misplaced while the optical fiber for the other rat had been damaged. These animals were therefore removed from analysis (*N* = 5). Key results from this experiment, including means and standard errors across subjects as well as the results of GEE analysis, are listed in Table 1.

**Table 1:** Mean and standard error of EEG signal properties in the theta and slow gamma bandwidths in the narrow ceramic chamber

Variable	Condition	CA1	CA1 *P*‐value	CA3	CA3 *P*‐value
Theta‐lo freq. (5–7 Hz)	Pre 6‐Hz stimulation	6.20 ± 0.024		6.12 ± 0.030	
	During 6‐Hz stimulation	6.04 ± 0.037	*P* = 0.001*	6.03 ± 0.014	*P* = 0.003*
Theta freq. (5–12 Hz)	Pre 6‐Hz stimulation	7.10 ± 0.13		7.20 ± 0.13	
	During 6‐Hz stimulation	7.35 ± 0.14	*P* = 0.105	6.78 ± 0.24	*P* = 0.006*
Theta freq. (5–12 Hz)	Pre 30‐Hz Stimulation	7.14 ± 0.15		7.29 ± 0.16	
	During 30‐Hz stimulation	7.62 ± 0.16	*P* = 0.012*	7.56 ± 0.17	*P* = 0.234
Theta amp. (5–12 Hz: A.U.)	Pre 6‐Hz stimulation	0.001016 ± 1.03 E‐05	*P* = 0.362	0.000985 ± 1.36 E‐05	*P* = 0.723
	During 6‐Hz stimulation	0.001005 ± 1.31 E‐05		0.000983 ± 8.75 E‐06	
Theta amp. (5–12 Hz: A.U.)	Pre 30‐Hz stimulation	0.001012 ± 1.55E‐05		0.0009679 ± 1.30E‐05	
	During 30‐Hz stimulation	0.000985 ± 8.70E‐06	*P* = 0.014*	0.0009570 ± 8.41E‐06	*P* = 0.08
Gamma amp. (28–32 Hz; A.U.)	Pre 30‐Hz stimulation	0.000884 ± 1.24E‐05		0.000887 ± 1.35E‐05	
	During 30‐Hz stimulation	0.000947 ± 1.38E‐05	*P* = 0.010*	0.000961 ± 2.51E‐05	*P* = 0.004*

Stimulations at 6 Hz tended not to affect theta amplitude but reduced theta frequency in CA3. Stimulations at 30 Hz caused corresponding increases in 30‐Hz amplitude in both regions while also influencing theta frequency and amplitude in CA1 (* = statistical significance; freq., frequency; amp., amplitude; A.U., arbitrary units for bandwidths of indicated frequency range).

**Figure 2:**
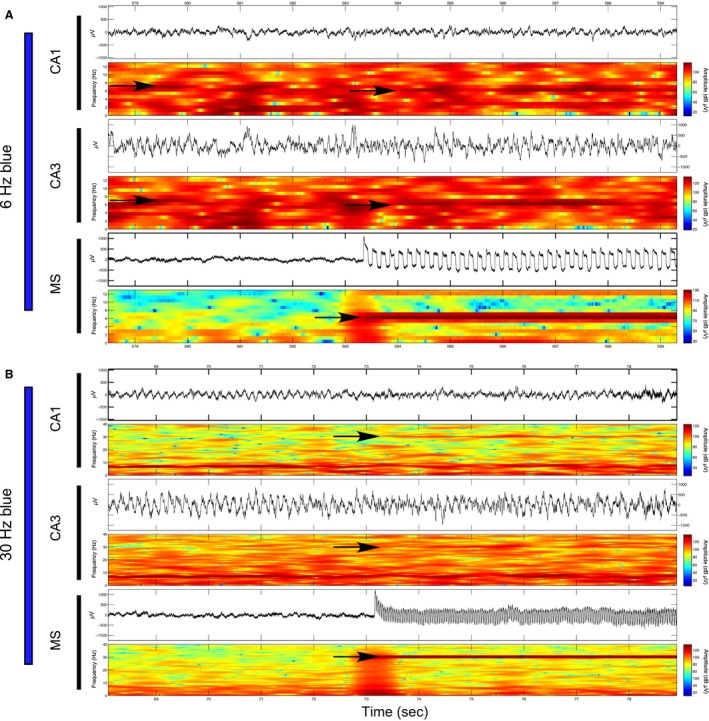
Examples of optical stimulation of the medial septum at 6 Hz and 30 Hz and corresponding influence over hippocampal oscillations in regions CA1 and CA3 while the rat sits in a narrow ceramic pot: (A) Raw EEG signal and spectrogram (top and bottom of each subsection) corresponding to the time periods approximately 6 sec before and 6 sec during 6‐Hz blue light stimulation of the medial septum (bottom), as well as CA3 (middle) and CA1 (top) fields of the dorsal hippocampus. Induced 6‐Hz oscillations are evident in the MS and the prestimulation theta frequency shifts from approximately 7 Hz in both CA1 and CA3 to 6 Hz during optical MS stimulation are indicated by black arrows; (B) As described in part A during a 30‐Hz blue light stimulation. Stimulations at 30 Hz in the MS cause corresponding increases at 30 Hz in both CA1 and CA3 that are indicated by black arrows.

**Figure 3:**
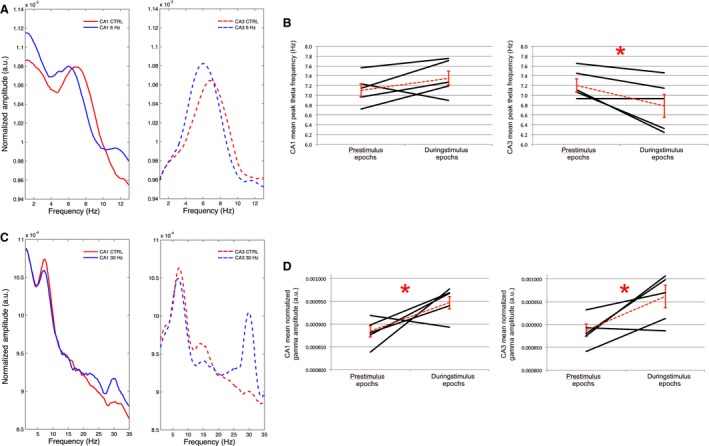
: EEG spectral data prior to and during multiple blue light stimulations at 6 Hz or 30 Hz: (A) Averaged amplitude spectrum for the five control epochs (red) and 5 MS stimulation epochs (blue) in CA1 (solid lines) and CA3 (dashed lines) for an individual rat. The average spectrum for this rat indicates frequency shifts at peak‐theta amplitude from approximately 7 Hz during prestimulation epochs to 6 Hz during 6‐Hz stimulation epochs in both CA1 and CA3. (B) Average and standard error of the mean peak‐theta frequency across rats (red dashed lines) as well as mean peak‐theta frequency for each individual rat (black lines; *N* = 5) during the five control and 6‐Hz stimulation epochs in CA1 (left) and CA3 (right). While the general trend across rats is for a nonsignificant increase in mean peak‐theta frequency in CA1 there is a significant decrease in mean peak‐theta frequency in CA3 where 2/5 animals demonstrate entrainment of theta frequency by MS stimulation at 6 Hz. (C) As in A, during averaged epochs before and during 30‐Hz blue light stimulation. Increases in the average signal amplitude are evident at 30 Hz during 30 Hz blue light stimulation in comparison to the average signal amplitude in this range in the prestimulation epochs. (D) Average and standard error of the mean gamma amplitude (28–32 Hz) across rats (red dashed lines) as well as mean gamma amplitude for each individual rat (black lines; *N* = 5) during the five control and 30‐Hz stimulation epochs in CA1 (left) and CA3 (right). The 30‐Hz blue light stimulation robustly alters signals in the 28–32 Hz bandwidths. (* = statistical significance).

Theta range stimulation at 6 Hz: Blue light stimulations at 6 Hz did not affect normalized theta amplitude in CA1 (*P* = 0.362) or CA3 (*P* = 0.723) in comparison with prestimulation epochs. However, as illustrated in spectrogram examples in Figure 2 (top) and EEG spectrums in Figure 3, we found that it was possible to entrain hippocampal theta oscillations through nonselective MS simulations. While 6‐Hz stimulations were not effective at altering CA1 theta frequency across rats (*P* = 0.105), this protocol did significantly decrease CA3 theta frequency in comparison with prestimulation epochs (*P* = 0.006) (Fig. 3B). From the five rats sampled, entrainment of CA3 theta frequency at approximately 6 Hz was evident for two rats while a decrease in mean frequency was observed for another two rats. No significant effect in theta frequency was found for one rat. If we limited our frequency analysis to the slower components of theta (5–7 Hz) that were closer to the stimulation frequency, we found significant decreases in mean peak frequency during stimulation epochs in both CA1 (*P* = 0.001) and CA3 (*P* = 0.003). While the more functionally relevant measures are in the full theta spectrum (5–12 Hz), we include this result as an indication that the effect of 6‐Hz MS stimulations on hippocampal theta oscillations may be different at both ends of theta spectrum. The direct entrainment of theta may depend on theta state and how the stimulation frequency interacts with intrinsic hippocampal theta frequencies across the bandwidth. We entertain this hypothesis in the following experiment in relation to animal speed during exploration of a novel open field environment.

Gamma range stimulation at 30 Hz: As illustrated by an individual example in Figure 2 (bottom) as well as across animals in Figure 3, optical MS stimulation at 30 Hz produces universal increases in the corresponding amplitude of hippocampal signals in the 28–32 Hz bandwidth in both CA1 (*P* = 0.010) and CA3 (*P* = 0.004) (Fig. 3C–D). Surprisingly, while stimulations spared theta oscillations in CA3, they perturbed theta oscillations in CA1 by significantly increasing mean theta frequency (*P* = 0.012) and significantly decreasing mean theta amplitude (*P* = 0.014) in comparison with prestimulation epochs. Therefore, in contrast to 6‐Hz MS stimulations, 30‐Hz stimulations more reliably entrained hippocampal oscillations in both hippocampal subfields.

### Novel environment

EEG signals were recorded while rats explored novel environments and received optical stimulation in the medial septum at 6 Hz and 30 Hz blue and yellow light control spectrums (*N* = 4). Key results from this experiment, including means and standard errors across subjects as well as the results of GEE analysis, are listed in Table 2.

**Table 2:** Mean and standard error of EEG signal properties of interest in the theta and slow gamma bandwidths during control stimulations as well as 6‐Hz and 30‐Hz blue light stimulation protocols during exploration of novel environments

Variable	Condition	CA1	CA1 *P*‐value	CA3	CA3 *P*‐value
Theta freq. (5–12 Hz)	Control stimulation	7.27 ± 0.20		7.46 ± 0.287	
	Blue 6‐Hz stimulation	7.49 ± 0.43	*P *= 0.613	7.61 ± 0.271	*P* = 0.421
Theta amp. (5–12 Hz: A.U.)	Control stimulation	0.001124 ± 6.30E‐06		0.00106 ± 1.54E‐05	
	Blue 6‐Hz stimulation	0.001087 ± 1.48E‐05	*P* = 0.007*	0.00105 ± 1.21E‐05	*P* = 0.005*
Gamma amp. (28–32 Hz; A.U.)	Control stimulation	0.000884 ± 7.35E‐07		0.000874 ± 3.084E‐06	
	Blue 30‐Hz stimulation	0.000947 ± 5.28E‐06	*P* < 0.001*	0.000924 ± 9.86E‐06	*P* < 0.001*

Averaged over the entire recording session, 6‐Hz blue light stimulations did not significantly alter theta frequency in either CA1 or CA3. Stimulations at 30 Hz caused corresponding increases in 30‐Hz amplitude in CA1 but not CA3 (* = statistical significance; freq., frequency; amp., amplitude; A.U., arbitrary units for bandwidths of indicated frequency range).

**Figure 4:**
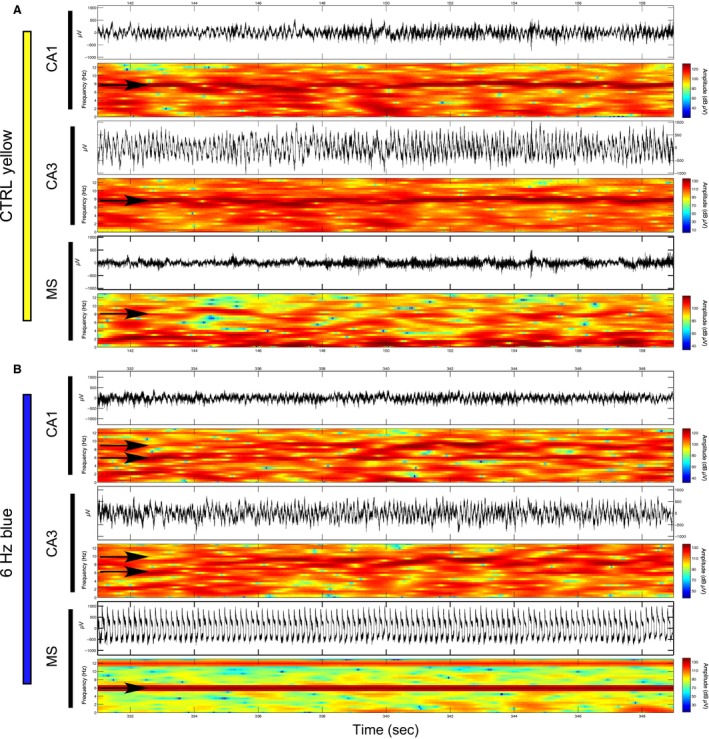
Examples of 6‐Hz optical stimulation of the medial septum and corresponding influence over hippocampal oscillations in regions CA1 and CA3 while the rat explores a novel environment: (A) Raw EEG signal and spectrogram (top and bottom of each subsection) corresponding to approximately 20 sec of 6‐Hz yellow light control stimulation of the medial septum (MS) (bottom), as well as regions CA3 (middle) and CA1 (top) of the dorsal hippocampus. The control stimulation does not cause a perceptible change in the oscillatory activity of either brain region; (B) Sample of approximately 20 sec in which 6‐Hz blue light stimulation in the MS induces a strong corresponding 6‐Hz oscillation while exploring a second novel environment. Theta range oscillations in CA1 and CA3 appear to toggle between 6 Hz and approximately 9 Hz (black arrows).

**Figure 5:**
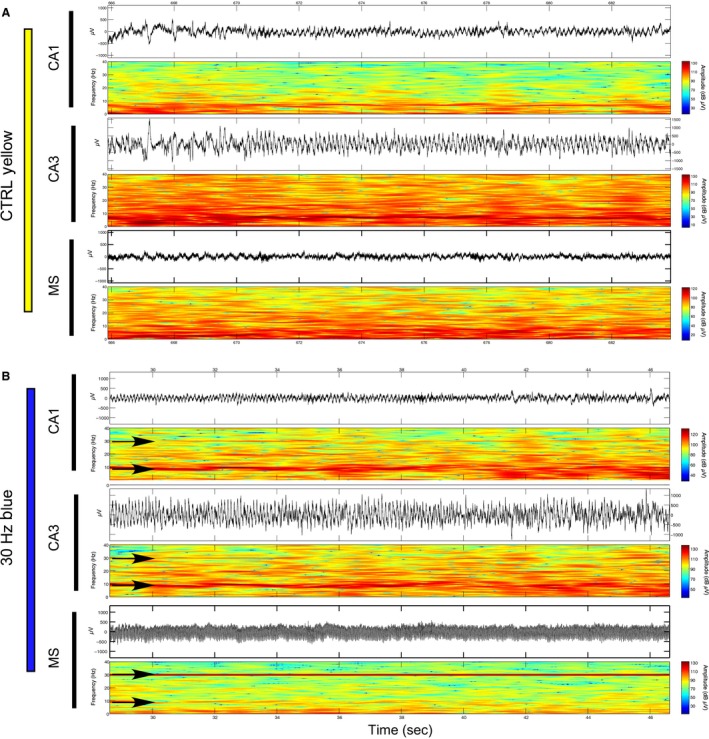
Examples of 30‐Hz optical stimulation of the medial septum and corresponding influence over hippocampal oscillations in regions CA1 and CA3 while the rat explores a novel environment: (A) Raw EEG signal and spectrogram (top and bottom of each subsection) corresponding to approximately 20 sec of 30‐Hz yellow light control stimulation of the medial septum (MS) (bottom), as well as regions CA3 (middle) and CA1 (top) of the dorsal hippocampus. The control stimulation does not cause a perceptible change in the oscillatory activity of either brain region, either at 30 Hz or within the theta range; (B) Sample of approximately 20 sec in which 30‐Hz blue light stimulation in the MS induces a strong 30‐Hz oscillation in the MS as well as corresponding 30‐Hz oscillations in CA1 and CA3 while exploring an additional novel environment.

**Figure 6:**
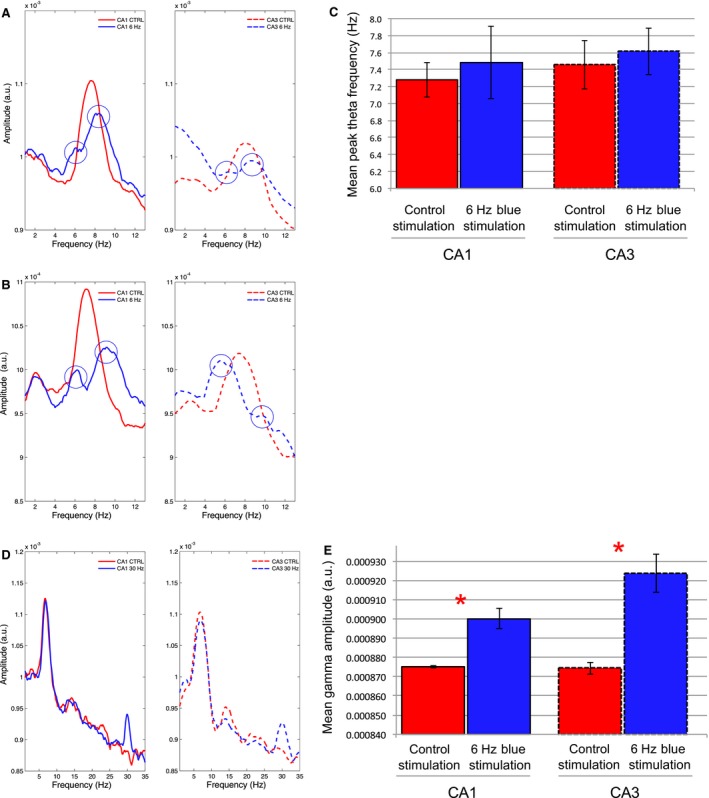
: Examples of the mean EEG spectrums during control‐yellow and blue light stimulations at 6 Hz or 30 Hz over the course of the entire recording session in which the rat explored novel environments (*N* = 4): (A‐B) The signal spectrums during 6‐Hz blue light MS stimulation (blue lines) from 2 animals (top and bottom) indicate a bisection of peak‐theta amplitude in comparison to 6‐Hz control stimulation (red lines) in both CA1 (solid lines at left) and CA3 (dashed lines at right). Two modes appear in the theta spectrum, one at approximately 6 Hz and the other at approximately 9–10 Hz; (C) The lack of a significant mean peak theta frequency effect across rats (*N* = 4) following 6‐Hz stimulation may be explained by the bisection of theta seen in A and B and the corresponding variability in theta frequency; (D) As in A–B, signal amplitude spectrums for an individual rat are compared between 30‐Hz blue light stimulation and 30 Hz yellow control light stimulation in the MS for both CA1 and CA3. The 30‐Hz stimulation again clearly increases the amplitude of the EEG signal in the 30 Hz range in both CA1 and CA3. (E) The mean and standard error of gamma amplitude (28–32 Hz) across rats. Significant increases in signal amplitude at 28–32 Hz were again found in correspondence to 30‐Hz blue light stimulation. (* = statistical significance).

Theta range stimulation at 6 Hz: While control stimulations had no significant effect on theta properties (Figs. 4, 5, 6), the effects of 6‐Hz blue light stimulation on theta frequency were even more complex during exploration of the novel environment than during limited mobility in the narrow ceramic chamber. GEE analysis revealed that while blue light stimulations at 6 Hz significantly decreased theta amplitude in both CA1 (*P* = 0.007) and CA3 (*P* = 0.005), they did not significantly affect the average peak theta frequency in either CA1 (*P* = 0.613) or CA3 (*P* = 0.421) in comparison with control stimulations (Fig. 6B). As illustrated by an example spectrogram in Figure 4, over the recording session, there was a tendency during 6‐Hz stimulations for theta signals to “toggle” between 6 Hz and approximately 9 Hz at approximately 0.5‐sec intervals.

Analysis of the averaged spectral data revealed a corresponding bisection of theta oscillations at these frequencies. Two separate examples illustrating this phenomenon in different rats are shown in Figure 6A.

**Figure 7:**
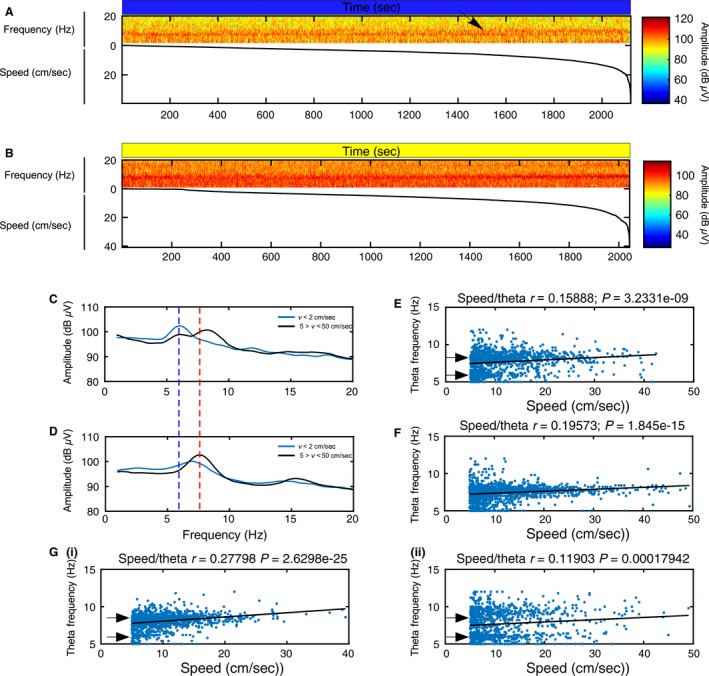
The relationship between animal speed and efficacy of 6‐Hz septal stimulations explains the bisection of theta frequency. (A) Speed sorted spectrogram showing signal amplitude during novel environment exploration and constant 6‐Hz blue light stimulation in relation to signal frequency (top) and animal speed sorted from slower to faster velocity (bottom). The plot shows that the largest signal amplitude in the theta range is consistently in the 6 Hz range until the animal moves at approximately 10 cm/sec (see black arrow). At this speed, the frequency in the theta range is consistently faster at approximately 9 Hz; (B) Speed sorted spectrogram showing signal amplitude during novel environment exploration and constant 6‐Hz yellow light control stimulation in relation to signal frequency (top) and animal speed sorted from slower to faster velocity (bottom). While the plot shows that there is some nonmovement‐related theta, the epochs with larger signal amplitude and faster frequencies in the theta range tend to occur with increases in animal speed; (C–D) Speed filtered amplitude spectrum during novel environment exploration sessions as in A and B with 6‐Hz blue light stimulation (C) and 6‐Hz yellow light stimulation (D). Black lines illustrate the mean spectrum when the rat's velocity (*v*) is between 5 and 50 cm/sec while the blue lines illustrate the mean spectrum when the rat's velocity (*v*) is less than 2 cm/sec. The plot in C shows that the theta frequency is more strongly driven at 6 Hz (dashed dark blue line) when the animal is moving less than 2 cm/sec and is bisected at either 6 Hz or toward 9 Hz when the animal is moving between 5 and 50 cm/sec. The theta peak in D during faster movements (dashed red line) falls in the normal theta range (~7.5 Hz). The faster component of the theta oscillation in C at around 9 Hz may be a network reaction to 6‐Hz stimulation; (E–F) Relationship between speed of movement and theta frequency (blue dots) throughout the 6‐Hz blue light stimulation (E) and 6‐Hz control stimulation sessions. The line of best fit (black lines) and the corresponding correlation coefficient between theta frequency and speed indicate a linear relationship in both sessions. The significant *r*‐value (*r* and *P*‐values indicated above plot) indicates that as animal speed increases, so does theta frequency. The plot in E shows that 6‐Hz oscillations are more evident in the theta signal when the animal moves less than 10 cm/sec while 9–10 Hz oscillations are more common when the animal moves faster than 10 cm/sec. (F) Theta frequency oscillations at 6 Hz or 9–10 Hz are less common during control stimulations. (G) Further examples of theta bisection during 6‐Hz blue light stimulation in the novel environment from two other animals (i and ii).

We examined the relationship between theta bisection and animal speed during active exploration and hypothesized that toggling between theta frequency extremes during 6‐Hz stimulation could occur due to speed‐related shifts in the MS influence over theta frequency. An individual example of the relationship between animal speed and theta toggling during stimulation is illustrated in Figure 7. If the animal tended to move at speeds greater than 5 cm/sec, theta frequency could be 6 or 9 Hz. If the animal moved slowly, at speeds less than 2 cm/sec, theta frequency was more likely to match stimulation frequency at 6 Hz (Fig. 7C). When the animal moved at speeds faster than 10 cm/sec, theta frequency was more likely to be approximately 9 Hz (Fig. 7E). In comparison, theta frequencies during control stimulations do not exhibit shifts at either of these extremes (Fig. 7D) and theta frequency during speeds greater than 5 cm/sec rarely approaches 9 Hz (Fig. 7F). Additional examples of the theta bisection phenomenon during 6‐Hz stimulation from two other animals are shown in Figure 7G.

Notably, while the 6‐Hz stimulation affected theta frequency in relation to animal speed, GEE analysis across all four animals revealed that stimulation did not alter the normalized correlation coefficient between animal speed and theta frequency during 6‐Hz blue light stimulations (mean = 0.1802 ± 0.0275) (Fig. 7E) and 6‐Hz yellow light control stimulations (mean = 0.152 ± 0.0123; *P* = 0.137) (Fig. 7F). We propose that theta bisection might represent a compensatory network reaction to 6‐Hz stimulations that maintains a linear relationship between theta frequency and speed. Moreover, this phenomenon may also explain why septal stimulation at theta frequency was rarely successful at directly entraining theta frequency when averaged over stimulation epochs lasting several seconds (Fig. 3B) or minutes (Fig. 6B).

Finally, the result of a paired t‐test indicated that there was no significant difference (*t* = 2.87; *P* = 0.0636) in the average animal speed between 6‐Hz blue light (mean = 11.9 ± 0.83 cm/sec) and yellow light control (mean = 13.65 ± 1.02 cm/sec) stimulation protocols. Therefore, although the 6‐Hz stimulations caused intermittent increases in theta frequency relative to control stimulation, this did not lead to an average increase in speed.

Taken together, our findings indicate that MS contributions to hippocampal theta may be greatest at slower speeds, and by inference, hippocampal theta may be more susceptible to MS manipulation during these epochs.

Gamma range stimulation at 30 Hz: As shown in the spectrogram for an individual rat in Figure 5, we again found corresponding increases in the amplitude of hippocampal signals in the 28–32 Hz range following 30‐Hz MS stimulations. GEE analysis revealed that 30‐Hz MS stimulation across rats again resulted in a significant increase in signal amplitude in both CA1 (*P* < 0.001) and CA3 (*P* < 0.001) (Figure 6E). Therefore, in both limited mobility and open field recording contexts, 30‐Hz blue light stimulation had a more systematic effect on the entrainment of hippocampal oscillations than 6‐Hz stimulations.

### T‐Maze

#### Behavioral results

**Figure 8:**
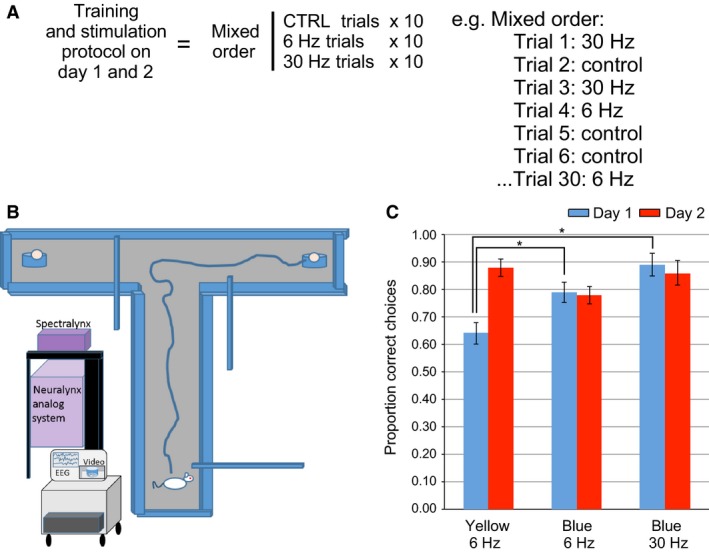
Alternating T‐maze experimental design and behavioral results. (A) Experimental design: There are two phases of T‐maze training on Day 1 and Day 2, where animals tended to make significantly more correct choices on Day 2. Each phase is composed of 30 trials of randomly ordered 6‐Hz yellow control, and blue light stimulations at 6 Hz or 30 Hz; (B) For testing, the rat is placed in the start area of the T‐maze. The gate is opened and the rat runs to end of the choice alley. For the first run, either the right or left arm is closed and the rat goes into the open arm with the food award. On the second trial, both arms are open and the rat is rewarded if it goes into the previously unbaited arm. This sequence is repeated for 30 test trials; (C) Statistical analysis demonstrated a significant increase in the percentage of correct choices during both 6‐Hz and 30‐Hz blue light stimulation on Day 1 of T‐maze training in comparison with 6‐Hz yellow light control stimulation. Performance on Day 2 across stimulation types did not differ significantly. Performance during control trials improved significantly between Day 1 and Day 2. (* = statistical significance).

As shown in the description of the T‐maze training protocol in Figure 8, stimulation protocols were randomized during training trials. On Day 1 of T‐maze training, GEE demonstrated a significant stimulation effect (*P* = 0.002) regarding the mean proportion of correct choices during blue light stimulations at both 6 Hz (0.79 ± 0.038) and 30 Hz (0.89 ± 0.041) trials in comparison with 6‐Hz yellow light control stimulation (0.64 ± 0.039). Significant performance improvements were found relative to control stimulations for both blue light stimulation frequencies at 6 Hz (*P* = 0.006) and 30 Hz (*P* = 0.001). Due to improved performance, no blue light stimulation effects were found with regard to performance in comparison with control stimulation on the second day of T‐maze training (*P* = 0.074). Although the mean proportion of correct trials during yellow light controls (0.88 ± 0.033) increased from Day 1 to Day 2, the mean proportion of correct trials during blue light stimulations at 6 Hz (0.78 ± 0.033) and 30 Hz (0.86 ± 0.046) stayed relatively constant. This result suggests that on the first day of training, both blue light stimulation protocols improved the probability of making a correct choice. However, as there was not a stimulation effect on Day 2, there was an apparent ceiling effect with regard to the degree to which stimulation could improve performance.

#### EEG results

EEG signals were recorded while rats learned and performed the T‐maze alternation task and received optical MS stimulation at 6 Hz and 30 Hz blue and yellow light control spectrums. A subset of animals whose hippocampal oscillations exhibited the clearest response to both stimulation protocols in the narrow chamber were selected for EEG analysis (*N* = 4). Analysis included both sample and choice trial types. Key results from this experiment, including means and standard errors across subjects as well as the results of GEE analysis, are listed in Table 3.

**Table 3:** Mean and standard error of EEG signal properties in the slow gamma bandwidth during 30‐Hz blue light stimulation during T‐maze training trials

Variable	Condition	CA1	CA1 *P*‐value	CA3	CA3 *P*‐value
Theta freq. (5–12 Hz: A.U.)	Control yellow stimulation	7.73 ± 0.113		7.85 ± 0.898	
	6‐Hz blue stimulation	7.94 ± 0.263	*P* = 0.147	8.03 ± 0.263	*P* = 0.812
	30‐Hz blue stimulation	7.97 ± 0.252	*P* = 0.08	8.07 ± 0.197	*P* = 0.103
Gamma amp. (28–32 Hz; A.U.)	Control yellow stimulation	0.0009024 ± 3.81E‐06		0.00091022 ± 9.06E‐06	
	30‐Hz blue stimulation	0.0009286 ± 9.30E‐06	*P* = 0.020*	0.0009564 ± 1.13E‐05	*P* < 0.001*

Stimulations at 30 Hz caused corresponding increases in 30 Hz amplitude in CA1 and CA3. Theta frequency was unaffected by either protocol (* = statistical significance; freq., frequency; amp., amplitude; A.U., arbitrary units for bandwidths of indicated frequency range).

**Figure 9:**
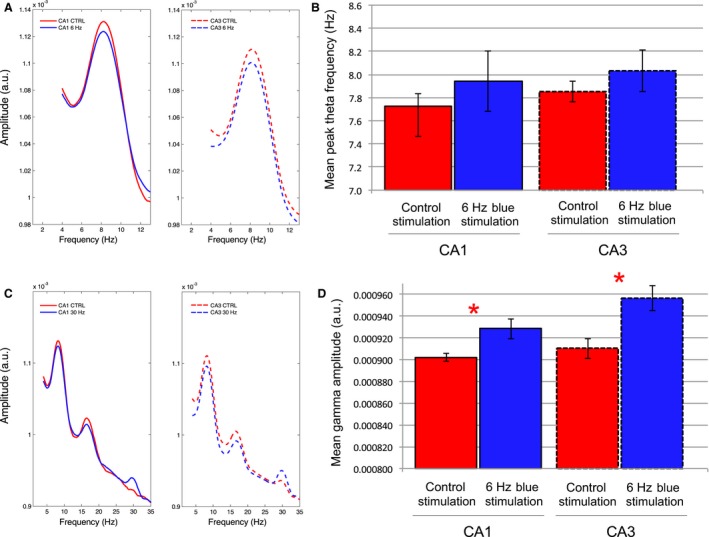
: Examples of the EEG spectrums during averaged stimulation epochs during multiple blue light stimulations at 6 Hz or 30 Hz or control yellow light stimulation at 6 Hz during runs down the choice arm of the T‐maze: (A) An example of the signal amplitude spectrums from an individual rat that shows the mean signal during 6‐Hz blue light stimulation (blue lines) and during control yellow light stimulation (red lines) in both CA1 (solid lines) and CA3 (dashed lines). (B) Mean and standard error of the peak‐theta frequency across animals (*N* = 4) indicates that there was no significant change during 6‐Hz blue light stimulations while the rats ran in the choice arm. (C) As in A, spectrum examples from an individual rat during averaged epochs of both control‐yellow and 30‐Hz blue light stimulation in the T‐maze choice arm. Increases in the average signal amplitude are again evident at 30 Hz during 30‐Hz blue light stimulation in comparison to the average signal amplitude during control stimulation. (D) Mean and standard error of gamma amplitude (28–32 Hz) across rats (*N* = 4). Significant increases in signal amplitude at 28–32 Hz were again found in correspondence to 30 Hz blue light stimulation. (* = statistical significance).

While both 6‐Hz and 30‐Hz stimulation protocols increased the probability of correct choices on Day 1 of T‐maze training, we did not find significant changes in theta frequency during either of these stimulation protocols while the animals moved down the choice alley (Fig. 9). MS stimulations at 6 Hz did not significantly change the mean peak‐theta frequency in either CA1 (*P* = 0.147) or CA3 (*P* = 0.812). As was the case in the previous protocols, the amplitude of signals in the 28–32 Hz bandwidth again increased significantly in response to 30‐Hz blue light stimulation in both CA1 (*P* = 0.020) and CA3 (*P* < 0.001).

Improved performance on Day 1 of the T‐maze training in response to 6‐ and 30‐Hz MS stimulations was therefore independent of changes in theta frequency. We discuss possible explanations for the improved Day 1 T‐maze performance in relation to MS stimulation below.

### Histology

We confirmed that there was an expression of the ChR2 in the medial septum (See Fig. 1) as well as accurate electrode placement in CA1 and CA3.

## Discussion

The primary goal of our study was to test the efficacy of nonselective optogenetic MS stimulation for effectively altering oscillations throughout the hippocampal circuit. This methodology has the potential for asking key questions regarding the role of hippocampal oscillations, particularly in the theta bandwidth, in the organization of cell assemblies that underpin learning and memory processes. We began with the assumption that optical control of MS oscillations would allow for indirect entrainment of the frequency of hippocampal oscillations. While MS optical stimulations at 30 Hz reliably entrain hippocampal oscillations, the determination of entrainment efficacy during stimulations at 6 Hz was much more complex. Stimulation protocols in a narrow chamber that allowed for limited mobility as well as a novel open field revealed that while it is possible for MS stimulation to entrain hippocampal theta oscillations, theta often interacts with the artificial inputs in a manner that largely depends on the animal's movement velocity. We also show for the first time that normal rats demonstrate improved learning on the first day of T‐maze training during 6‐ or 30‐Hz stimulations. While the underlying mechanisms of this improved performance remain unclear, the study demonstrates that nonselecitve optogenetic stimulation can influence network activity, learning and memory.

### Efficacy of MS theta range stimulation is yoked to movement

Experiments in the narrow chamber showed that, on average, the 6‐Hz MS stimulations were not effective at directly entraining CA1 oscillations. However, this protocol was more effective at lowering average CA3 theta frequency across rats, and in two of these cases entrained theta at 6 Hz. While the differences in the susceptibility of both subfields to MS stimulation entrainment was surprising, the results suggested that there may be another variable mitigating the MS entrainment of hippocampal oscillations. While the narrow chamber limited the animal's movement, it was still possible for the animal to move and rear. We hypothesized that movement could be the missing variable that influenced MS control of hippocampal theta and therefore carried out the second series of stimulation experiments during exploration of a novel open field. Averaged over 20 min of stimulation, MS entrainment of theta oscillations across animals appeared to be even less effective in the open field than in the narrow chamber. However, analysis of the mean spectral data for individual animals revealed a novel phenomenon. During 6‐Hz MS stimulations, theta was bisected into a bimodal amplitude distribution with peaks at the 6‐Hz stimulation frequency and at 9–10 Hz. Speed/theta analysis uncovered a tendency for theta frequency to be entrained at 6 Hz when the animals moved less than 2 cm/sec, to toggle between 6 and 9 Hz between 5 and 10 cm/sec, and to oscillate at approximately 10 Hz when they moved greater than 10 cm/sec.

Theta bisection provides two points to consider. The first is that MS contribution to theta frequency may be greatest when the rats move at slower speeds. This may explain the greater efficacy of MS entrainment of hippocampal theta in those epochs and the difficulty we had in directly entraining theta frequency during stimulation periods that lasted many seconds or many minutes. That theta is more readily “sculpted” by MS stimulation during slow speeds or immobility is in line with previous work that has shown the importance of the septo‐hippocampal axis in relation to processing behavioral state changes. The second point to consider is the origin of the faster theta component at 9–10 Hz. One possibility is that it could stem from the merging of the artificial 6‐Hz oscillation with intrinsic theta oscillations at 8 Hz. However, beat frequencies from these two oscillations would be more likely to create an irregular, slower oscillation than a faster one. We propose that the 9–10 Hz theta component represents a homeostatic network correction that serves to maintain an average frequency within the theta band as well as a linear speed–theta relationship. Importantly, this occurs without alterations to the mean animal speed during stimulation.

Hippocampal theta rhythm has multiple generators and, while important, is dependent on more than the reciprocal connectivity with the medial septum and other subcortical structures. One of these complimentary hippocampal theta generators, also influenced by inputs from the medial septum, is the entorhinal cortex (Brandon et al. 2011). Recent work has shown that it is possible to dissociate the linear speed relationship of both cell activity and local theta oscillations in the entorhinal cortex, where only the firing rate activity of cells in relation to speed is strengthened following inactivation of the medial septum (Hinman et al., 2016). It is therefore possible that entorhinal activity can serve both as an error corrector (Ketz et al. 2013) and a path integrator (McNaughton et al. 2006bb) in the speed/distance calculation circuit during 6‐Hz MS stimulation and influence hippocampal theta oscillations to maintain a linear speed/theta relationship. Further stimulation experiments at both faster and slower theta frequencies will be necessary to confirm this hypothesis.

### MS stimulation improves performance

Perhaps, the most surprising result of our study was that both 6‐Hz and 30‐Hz optical stimulation protocols, in comparison with control stimulations, were found to increase the likelihood of making a correct choice on the first day of T‐maze training. While the stimulations at 30 Hz reliably produce corresponding increases in the amplitude of 28–32 Hz signals, within the slow gamma bandwidth, the resultant effects by 6‐Hz stimulations were unclear. Unlike the other two behavioral contexts, there was no detectable change in the frequency of theta oscillations in CA1 or CA3 during 6‐Hz blue light stimulations. This may be due to the nature of the task, considering the short time period involved in moving through the center arm where the animals presumably made their alternation decisions. Ultimately, with regard to both stimulation protocols, the mechanism by which optogenetic stimulation enhanced alternation decisions in the early acquisition of T‐maze performance is unknown. Perhaps, the clearest result from this experiment is that MS stimulation protocols did not hinder performance of the T‐maze task during the first day of training.

Although typically carried out with either lesions or temporary inactivation of the medial septum, there is a growing literature in relation to the use of artificial MS theta stimulation (ATS) in rats as a type of neuroprosthetic that enhances learning and memory in disease models where intrinsic theta oscillations are compromised (Turnbull et al. 1994; McNaughton et al. 2006aa; Shirvalkar et al. 2010; Lipponen et al. 2012). However, this literature has shown mixed results on the ability to restore or improve memory in animals with either permanent or temporary loss of MS activity. Modest degrees of improvement have been found in spatial working memory tasks using ATS protocols (Turnbull et al. 1994; McNaughton et al. 2006aa), or theta burst stimulation protocols (Shirvalkar et al. 2010). However, another study has found that even though ATS in the medial septum could restore hippocampal theta oscillations in fimbria fornix‐lesioned rats, this treatment was more likely to disrupt the encoding of contextual fear responses in these animals. Moreover, ATS was found to result in a significant impairment in contextual fear conditioning in sham‐lesioned animals (Lipponen et al. 2012). This has led to the suggestion that hippocampal theta may be more relevant for working memory tasks and memory retrieval than the encoding of information into long‐term memory (Lipponen et al. 2012).

An alternative interpretation for our results is that stimulation might bias the rats toward a nonhippocampal‐dependent strategy in the early training trials. This idea is supported by previous experiments in the Multiple Parallel Memory Systems (MPMS) literature where lesions or temporary inactivation of specific brain structures improve behavioral performance by limiting strategy‐dependent competition between brain regions (Packard and McGaugh 1996; White and McDonald 2002). Future experiments can take account for this possibility through further behavioral testing that examines the effects of stimulation on the tendency to use motor response strategies versus hippocampal‐dependent place‐based strategies with regard to T‐maze performance.

As improved performance was independent of theta properties, 30‐Hz MS stimulations may provide more relevant effects as they were found to increase the amplitude of oscillations within the slow gamma range that have been proposed to be associated with the routing of information between CA1 and CA3 as well as memory recall (Colgin et al. 2009; Shirvalkar et al. 2010). This interpretation should also be considered in light of the observation that performance enhancements were slightly better following 30‐Hz stimulation than 6‐Hz stimulations, on both the first and second day of T‐maze training.

A final alternative interpretation is that improved performance in relation to our stimulation protocols could reflect increased levels of acetylcholine from the medial septum to the hippocampus and other components of the limbic circuit. Alterations in levels of acetylcholine could affect levels of neural plasticity in complex ways. The timing of the arrival of acetylcholine from the medial septum at stratum oriens, in relation to CA3 inputs from the Schaffer collaterals, can promote nicotinic receptor‐dependent long‐term potentiation and short‐term depression or muscarinic receptor‐dependent long‐term potentiation (Gu and Yakel 2011; Yakel 2014). In order to tease apart the variables that might improve learning after medial septum stimulation, we ultimately need to dissociate the biophysical aspects of the tuning of hippocampal oscillations from the pharmacological influences of GABA and acetylcholine on hippocampal plasticity. Ideally, this could be accomplished through combined electrophysiological, optogenetic, and amperometry techniques (Howe et al. 2013). Replicating the experiment with intraventricular or systemic injections of scopolamine or other anticholinergic drugs (Brazhnik et al. 2003) would also determine the putative role of acetylcholine in learning enhancements associated with MS optical stimulation.

### Selective versus nonselective optogenetics

The key advantages of optogenetics as a research tool are its temporal accuracy as well as its ability to be selective for specific cell types when used in combination with transgenic rodent lines (Tye and Deisseroth 2012). For example, the ability to selectively activate or inactivate specific cell types has allowed for the exploration of the notion that rhythmic bursts of cholinergic and GABAergic cells entrain hippocampal neurons, whose corresponding rhythmic excitation and inhibition generate at least one component of the theta rhythm (Stewart and Fox, 1990). In direct continuance of this conceptualization, cell‐type selective optogenetics has shown that septal cholinergic and GABAergic cells have contrasting influences over hippocampal theta oscillations that are dependent on levels of motor behavior and exploration (Vandecasteele et al. 2014; Bender et al. 2015; Mamad et al. 2015). Optical stimulation of septal cholinergic cells in anesthetized mice blocked hippocampal sharp wave ripples (Vandecasteele et al. 2014) while stimulation in inactive but awake mice was found to increase the amplitude of hippocampal theta (Mamad et al. 2015). Notably, the effects of optical stimulation of septal cholinergic cells on hippocampal theta was more effective during anesthesia and less effective in freely moving mice (Vandecasteele et al. 2014; Mamad et al. 2015), although it was found to increase the ratio of theta to slow oscillations (Vandecasteele et al. 2014) and alter the organization of hippocampal place cells (Mamad et al. 2015). In a complimentary fashion, imaging studies have shown that GABAergic axon terminals in CA1 emanating from the medial septum are most active during locomotion and salient sensory events and abolished during anesthesia (Kaifosh et al. 2013). In addition, through optical stimulation of the lateral septum, Bender et al. (2015) found that they could reliably entrain hippocampal theta. Resultant theta oscillations not only matched optical stimulation frequencies between 5 and 12 Hz but also produced a corresponding decrease in both the animal's speed and speed variability. While receiving less attention than GABAergic and cholinergic cells, glutamatergic cells in the medial septum have also been shown to regulate the modulation of CA1 pyramidal cell activity in relation to animal speed (Fuhrmann et al. 2015).

Our results using optogenetic stimulation in wild‐type rats have had similar findings to studies that selectively stimulated specific cell types in the medial septum. We also show a relationship between the efficacy of MS stimulation in relation to animal behavior and, in particular, animal speed (Vandecasteele et al. 2014; Bender et al. 2015; Mamad et al. 2015). A major implication of our results is that it is possible to address physiological questions related to the properties of hippocampal oscillations using optogenetic MS stimulation techniques without the use of transgenic rodent lines. A caveat to this suggestion is that the efficacy of this approach largely depends on the animal's behavior. This caveat is largely similar to the results of experiments with selective optogenetics, where the ability of MS stimulation to entrain hippocampal theta oscillations is more effective when the animals are moving slowly or are at rest. A second caveat is that MS stimulation has an effect on learning and memory that is independent of theta frequency alterations. This demands that alternative behavioral and neuromodulatory influences, particularly of acetylcholine, on learning and memory should be considered in future MS stimulation experiments.

## Conflict of interest

We declare no conflicts of interest.
